# Detection of fusidic acid resistance determinants among *Staphylococcus aureus* isolates causing skin and soft tissue infections from a tertiary care centre in Chennai, South India

**DOI:** 10.1186/1471-2334-12-S1-P45

**Published:** 2012-05-04

**Authors:** A Nagarajan, K Arunkumar, M Saravanan, G Sivakumar, Padma Krishnan

**Affiliations:** 1 Dept. of Microbiology, Dr. ALM PG Institute of Basic Medical Sciences, University of Madras, Chennai-600113, India; 2 Rajiv Gandhi Govt. General Hospital and Madras Medical College, Chennai-600003, India

## Background

Fusidic acid (FA)- an inhibitor of protein synthesis has been used for treating superficial and some systemic infections caused by *Staphylococcus aureus*. Fusidic acid resistance in *S. aureus* has been reported throughout the world with prevalence ranging from 0.5% to 50% and is due to i) point mutation in bacterial *fusA* or *fusE* gene and ii) by acquired FA resistance determinants *fusB*, *C* and *D*. Indian report of fusidic acid resistant *S. aureus* (FRSA) is based on phenotypic detection. Hence, this study was done to detect acquired FA resistance determinants.

## Methods

The study included 54 isolates of *S. aureus* collected from skin infections between Jan to Mar 2011 from a tertiary hospital in Chennai. MRSA was screened by cefoxitin disc diffusion method and PVL-MRSA detection was done by multiplex-PCR. FA resistance was screened by disc diffusion method and acquired resistance determinants were detected by multiplex-PCR.

## Results

Of the 54 *S. aureus* isolates, 32(59.2%) were found to be MRSA. A total of 13(24.1%) isolates were found to carry *pvl* gene of which 4 were MRSA. Two of the 54(3.7%) isolates were found to be FRSA and harbored *fus*C gene. Both FRSA isolates were from non-hospitalized patients and they were using FA for topical treatment.

## Conclusion

We report for the first time in India the presence of acquired FA resistant determinant *fus*C gene in community isolate of methicillin susceptible *S. aureus*. Indiscriminate use of FA needs to be avoided to prevent the emergence of FRSA.

